# Pretend play as the space for development of self-regulation: cultural-historical perspective

**DOI:** 10.3389/fpsyg.2023.1186512

**Published:** 2023-12-19

**Authors:** Milda Bredikyte, Agne Brandisauskiene

**Affiliations:** Educational Research Institute, Education Academy, Vytautas Magnus University, Kaunas, Lithuania

**Keywords:** pretend play, self-regulation, child’s play and self-regulation (CP&SR) checklist, cultural-historical theory of development, self-initiated and self-organized imaginative play

## Abstract

Self-regulated behavior is a prerequisite for learning and success in life. Considerable research confirms that mature forms of play support the development of self-regulation in the early years. This study explores the relationship between (3–6-year-olds) children’s pretend play and self-regulation skills. Teachers filled out a *child’s play and self-regulation checklist* evaluating the level of children’s play and self-regulation skills. The findings revealed that the levels of children’s play and self-regulation skills are statistically significantly linked: the better the child performs an assumed role in play activity, the higher the level of their self-regulation. The results also suggest that a child’s playing skills, gender, and age predict children’s self-regulation skills manifested in play activity.

## 1 Introduction

There has been a shift in educational thinking and practice in recent decades. Advances in research in several scientific fields brought the understanding that how well students do in school and later in life can be determined by how well they self-regulate (Shonkoff and Phillips, 2000; Duckworth and Seligman, 2005; Blair and Diamond, 2008; Shanker, 2013). As a result, researchers are increasingly focusing on self-regulation in young children. For example, Whitebread et al. (2009), Robson (2010), and Whitebread and Pino-Pasternak (2010) investigated the relationship between self-regulation and metacognition in young children. These researchers argue that we can see many signs of metacognitive and self-regulatory behavior by analyzing video recordings of young children’s activities. Such a methodological approach can benefit further research (Whitebread et al., 2009). However, as Robson (2010) points out, research on the emergence and development of early metacognition and self-regulation in young children is at the earliest stages of development. The lack of suitable research strategies, methods, and tools might be the main obstacle to studying these processes. Perry (2019) also emphasizes that scientists studying the development of self-regulation in young children may face the challenge of presenting authentic developmental tasks that would provide opportunities for young children to demonstrate their self-regulation abilities. “Much of the research involving young children is carried out in laboratories using standardized measures that are poor reflections of children’s self-regulation in their daily activities” (Perry, 2019, p. 332).

In their study, Vasseleu et al. (2022) seek to find how educators understand children’s self-regulation, its development, and practices that support the development of self-regulation in children. The results of this study (Vasseleu et al., 2022) show that teachers tend to concentrate on instances of dysregulation and focus mainly on cases of poor self-regulation, overlooking the rest of the children. However, as emphasized in much recent research (Robson et al., 2020), self-regulation skills are vital to academic success and foundational to successful participation in society (Colliver et al., 2022). Vasseleu et al. (2022) concluded that teachers’ professional development needs to focus on seeing all children and finding ways to facilitate and support the development of all children’s self-regulation.

Several studies report that self-regulation skills are often learned through high-quality pretend play (Berk and Meyers, 2013). Veraksa et al. (2022), exploring the relationships between play and executive functions, point out that play is one of the child’s main forms of activity, where he or she masters new forms of behavior, learns to interact with others, and develops imagination and self-regulation skills. A recent longitudinal study from Australia (Colliver et al., 2022) confirmed that the time spent in free, unstructured play at ages 2–3 and 4–5 years predicted self-regulation abilities 2 years later.

Many studies highlight the role that play has in a child’s social-emotional learning (Posner and Rothbart, 2000; Elias and Berk, 2002; Berk et al., 2006; Bodrova and Leong, 2007; Sherwood and Reifel, 2010; Kelly and Hammond, 2011; Hoffmann and Russ, 2012; Berk and Meyers, 2013; Diamond, 2013; Stagnitti et al., 2016; Walker et al., 2020). However, research on the impact of play on children’s development and learning is subject to criticism, much of which, as in self-regulation research, relates to the choice of appropriate strategies, methods, and tools.

The debate on the impact of social pretend play on the development and learning of preschoolers (Lillard et al., 2013) emerged over a decade ago, highlighting the problems of play research. This debate (Bergen, 2013; Sutherland and Friedman, 2013; Walker and Gopnik, 2013; Weisberg et al., 2013; Weisberg, 2015) revealed the understanding of how complex the phenomenon of play is and how we are sorely lacking in theoretical explanations and adequate research methods to study and analyze it. The situation in play research has remained relatively the same; the impact of play on separate areas of child development or learning continues to be studied through experimental targeted interventions (Richard et al., 2021; Jaggy et al., 2023).

As a result, in the background, an intense debate continues about what is more important in early childhood: to play more or start early academic teaching and learning. Studies by many scientists have shown that the recent rise of academic pressure during the early years often contributes to lower levels of the quantity and quality of pretend play (Becker et al., 2014), regardless of the general claim that these are the years when children need the most play-based learning opportunities.

As if seeking to resolve this contradiction more recently, a middle-ground perspective has gained popularity, with play as a joint activity of children and adults. This perspective takes many forms, such as “conceptual play” (Fleer, 2011, 2015); “playful curriculum” (Sim, 2015); “guided play” (Weisberg et al., 2016); “purposefully-framed play” (Edwards, 2017); “playful pedagogies” (Broadhead, 2018); “intentional teaching” (Lewis et al., 2019); and “purposeful play” (Moedt and Holmes, 2020). The very general idea of this perspective is that children and adults meet through play to build knowledge together. The knowledge is often but not necessarily understood as children’s scientific concepts, language, literacy, mathematics, and social skills.

To avoid ambiguity, we clearly define the theoretical approach of our research and use the concepts of cultural-historical theory to define play and self-regulation. In cultural-historical terms, the forms of play that claim their goal content knowledge for children are defined as *didactic play*. It is the most appropriate form of teaching for young children. However, this is not the *self-initiated* and *self-organized imaginative play* that, according to Vygotsky (2016), creates the zone of proximal development for the child and is potentially the most stimulating for developing children’s self-regulation.

In the cultural-historical frame, dynamically developing social imaginary play is the child’s first independent activity system, which they feel their own (Hakkarainen, 1999). Because it is a jointly created dynamic system, it binds children together through the play roles, forces them to follow the rules of the roles, and prevents them from falling out of the play quickly. Players are placed in a situation that begins to regulate their behavior, and even without knowing the exact rules of play, they quickly figure them out through play actions. If the play activity interests the children, they mobilize all their efforts to develop it. Perhaps it is this kind of involvement that Vygotsky described: “[I]n play, the child is always above his average age, above his daily behavior; [.] play contains all developmental tendencies in a condensed form” (Vygotsky, 2016, p. 18).

From a developmental point of view, child-initiated play is a precious activity in which the child’s interests, motives, and abilities are revealed. By creating meaningful play, the child mobilizes their full potential and often goes beyond everyday behavior. The professional educator must be able to observe, monitor, support, and evaluate the quality of the child’s play and participate in children’s play as a play partner without taking the lead or taking over the children’s play idea. Teacher’s participation is sometimes beneficial for the children as he/she can demonstrate new play strategies and ways to extend and enrich the children’s initiatives and ideas. In this case, the children learn to play from the adult. However, our research shows (Brėdikytė, 2022) that children and teachers are learning to play from each other when participating in collaboratively created play activities.

Another pedagogical aspect is that the teacher can easily see the children’s interest in the play and use it later to plan and organize explorative and intentional teaching episodes in a *didactic play* format. When the teacher offers children meaningful learning activities as a follow-up to their play, they inspire and enrich the children’s further play and learning. This continuous flow of initiatives from child-initiated play toward meaningful teacher-led learning, then back to children’s independent play, could be the basis for a successful preschool pedagogy (Hakkarainen et al., 2015; Brėdikytė and Hakkarainen, 2017).

As researchers in the cultural-historical paradigm, we emphasize imaginative role-play as a leading activity for child development. For us, imaginary play is the process of creating something new—a movement, an object, an idea, a sense, or a meaning. A child participating in this creative process develops new psychological formations, which lead them to a qualitatively new level of functioning. From our point of view, this is the primary purpose of *independent, self-organized* play activity. Mature forms of imaginary play develop general abilities in young children: (1) general creativity (creative improvisation, symbolization); (2) motivation; (3) imagination; (4) volition, voluntary behavior (self-regulation); (5) understanding of the other person’s point of view; (6) orientation toward the universal meanings of human life, relationships, and activities. Vygotsky called these abilities “psychological functions,” and the terms more familiar to readers may be: “cognitive abilities” (Dickinson et al., 2016), “cognitive tools” (Egan, 1997), or tools of mind (Bodrova and Leong, 2007). In addition, children acquire specific academic skills and knowledge more easily during play, but this is instead a by-product and not the primary goal of play activity.

The ambiguous explanations of the significance of childhood play and the resulting diversity of educational practices have inspired us to develop a theoretically based tool to help teachers observe and assess children’s pretend play and self-regulation and monitor their developmental dynamics. Our initial assumption that high-level pretend play contributes to the development of children’s self-regulation helped us to formulate the primary goal of this research project:

• To explore the relationship between the level of children’s pretend play and self-regulation skills.

This study extends the cultural-historical line of research on play, proposing to look at the development of higher psychological functions as social practices. According to Vygotsky and his followers, imaginative play is the activity that creates the optimal conditions for developing these functions. Fleer et al. (2017) argue that such a view is more “pedagogically productive” as it “speaks directly to teachers” practice. Children’s independent play is a common social practice in early childhood classrooms.

This study is distinctive among other studies. It explores *self-regulation* as *a social practice* that develops in close kinship with children’s *self-initiated* and *self-organized imaginative play*. A cultural-historical perspective on child development informed the choice.

In the following paragraphs, we will define the main theoretical concepts, describe the tool–*child’s play and self-regulation* (CP&SR) checklist (Supplementary Appendix 1) used for data collection, and explain its parameters. We will introduce the participants in the study, data collection, and analysis processes, discuss the results, and conclude with the implications for further research and practice.

## 2 The theoretical framework of the research

Our approach to play and child development is based on cultural-historical theory, especially on Vygotsky (2016) and El’konin (2005a,b) writings on play and early development. The main ideas about the child’s cultural development and the role of the social environment as the source of development inform our understanding of play, its functions in child development, and how we evaluate play.

In the cultural-historical tradition, the closest concept to self-regulation is *voluntary behavior* or *arbitrariness* (*произвольное* *поведение*, *произвольность*, *proizvol’noe povedenie*, *proizvol’nost’*). Vygotsky has shown that arbitrariness is not only a social formation by its origin and content but also by its mechanisms. The child does not adapt to the requirements of society and culture; it initially develops within this society and culture as an active participant.

At an early age, before three, the child is not free from the stimuli around him. Voluntary behavior allows the child to overcome dependence on what they perceive in the “here and now.” Voluntary behavior is the child’s ability to plan, manage, and evaluate his or her activities and behavior purposefully and consciously. The development of arbitrariness is among the main goals of early childhood education and an important criterion of school readiness.

One of the most prominent experts on self-regulation, Shanker (2013), proposed a detailed description of developed self-regulation, which aligns with the cultural-historical approach. Drawing on the insights of different scholars, he defined “self-regulation as the ability to: (1) attain, maintain, and change one’s level of energy to match the demands of the task or situation; (2) monitor, evaluate, and modify one’s emotions; (3) sustain and shift one’s attention when necessary and ignore distractions; (4) understand both the meaning of a variety of social interactions and how to engage in them in a sustained way; (5) connect with and care about what others are thinking and feeling—to empathize and act accordingly.” (Shanker, 2013, xii).

Shanker (2010, 2013) suggests a dynamic systems view of self-regulation and claims that early experiences play a critical role in the organization of the human brain. Because babies are born with a limited capacity to control their impulses, their self-regulation develops by being regulated by caregivers. Self-regulation develops throughout childhood, adolescence, and young adulthood as the challenges to which the child is exposed increase. The most important early experiences are the child’s interactions with their closest adults and peers. At a certain point, the child must start to monitor his behavior and activities. We are interested in how such a transition happens through a child’s participation in collaborative pretend role play.

Rephrasing Vygotsky (2016), a child’s most significant power of self-regulation arises in play. At all stages of play, a child is in a state of conflict between the rules of the play and spontaneous actions. In play, the child acts against his immediate wishes and at the limit of his willpower. Continuing Vygotsky’s and Elkonin’s line in play research, Smirnova (1998) analyzed the psychological mechanism of how the play role and play plot (sjuzhet) transform the child’s behavior. In her opinion, play does this through two main aspects: the child’s motivation and the execution of the role. The motivation for play lies in the very process of performing the activity.

Moreover, the execution of a desirable role is emotionally attractive to the child. “Correct” implementation of a role and plot becomes a motive of their activity. Having obtained a desired role, the child is forced to act according to the rules of this role. Why suddenly the child voluntarily obeys these rules? In play, the rule is detached from the child, as if it is taken outside and directed at the role. In play, the child regulates the “other,” not oneself. The in-role position helps the child to monitor and control their behavior through the “correct” execution of the role.

Thus, role-play of the preschooler in a natural form combines two necessary conditions for the development of voluntary self-regulated action–an increase in motivation and self-awareness, assuming a certain amount of reflexivity in the child.

## 3 Methodology

We have investigated the relationship between the child’s play and self-regulation skills. In the following, we will present the study design, describe the participants in the study, and explain how the data was collected and analyzed.

### 3.1 Participants

The study was conducted in seven daycare centers in Vilnius, Lithuania. The study involved 454 children (50% girls and 50% boys) aged 1.9–7 years (*M* = 56.64 months; SD = 1.26). Detailed information on the age of the children is given in Table 1.

**TABLE 1:** Age characteristics of the participants in the study’s first phase.

Age of the children	*N*	Min	Max	Mean	Std. deviation
2–3 years old	93	1.9	3.4	2.808	0.3979
3–5 years old	216	3.5	5.3	4.473	0.5339
5–7 years old	139	5.5	7.0	6.117	0.3614

### 3.2 Ethical considerations

The research was conducted following the ethical rules of the Helsinki Declaration. Verbal consent was obtained from the principal of each ECEC school before the study. Participating teachers sign informed consent. All parents were informed about the study and gave their children written permission to participate. ECEC teachers and children participated in the study voluntarily.

### 3.3 Data collection

At the beginning of the study, we had to choose how to collect genuine data on children’s play and self-regulation. We had to choose who would collect the data: university researchers or daycare teachers working with children regularly and knowing them well. Our conscious decision was to choose the teachers instead of the researchers, who would enter the classrooms as strangers and disturb ordinary life. It was important for this study to obtain the data from playing children within their familiar environment. All 75 teachers received initial training on children’s play. In addition, before the data collection, the researchers visited every daycare center, met with all participating teachers, presented the checklist, and explained the parameters in detail. During the meetings, teachers had to evaluate one child from their classroom (a pilot evaluation). After the training, the teachers were asked to observe six children in their classroom for a week and fill out the *child’s play and self-regulation* (CP&SR) checklist. Nevertheless, ensuring that all teachers assessing children’s play and self-regulation skills had the same conceptual understanding of these phenomena was essential. However, not all teachers were able to attend our training sessions. When this happened, extremely low Cohen’s d (Kappa) values were found (e.g., 0.209, 0.379). The analysis did not include these teachers’ ratings because they were unreliable. We describe this in more detail in the limitations section.

Most of the teachers in the study worked with mixed age groups of children (1.9–7 years), and many could select children of different ages for the assessment. The requirements for selecting the children for the study were to select three boys and three girls to select children of different ages and play skills if given the opportunity.

### 3.4 Research tools

The *child’s play and self-regulation* (CP&SR) checklist (Brėdikytė et al., 2015) was used to evaluate children’s performance. The checklist consists of two parts. The first part of the checklist was meant to evaluate a child’s play level.

A group of psychologists from the Center for Psychological and Pedagogical Expertise of Play and Toys of the Moscow State University of Psychology and Education have developed an original observation protocol for evaluating the level of children’s free play (Smirnova and Ryabkova, 2013; Ryabkova et al., 2017). We developed our tool in close collaboration with the researchers from this center. It was essential that the tool was theoretically sound and reflected the key structural components of pretend play. Our checklist contains all the same parameters as the observation protocol of our colleagues from Moscow, but we organized them into a convenient format for teachers. Our goal was to develop and validate an observation checklist that assesses pretend play level and self-regulation and provides the teacher with an instrument that would help them gain a deeper understanding of the development of a child’s play on the one hand and, at the same time, monitor the dynamics of the play and self-regulation skills in children.

Our (CP&SR) checklist includes seven structural components of play activity proposed by El’konin (1999a,b): (1) play objects, (2) self-position of the child, (3) interactions with a play partner, (4) play space, (5) play actions, (6) the play plot (sjuzhet), and (7) the main content of play. Each parameter of play observation is ranked on four different levels (from simple activity to more complex), e.g., the self-position of the player:

– Child has no role;
– Child has a role but does not keep to the rules of the role or is inconsistent;
– Child has a role and keeps to the rules of the role;
– Child is flexible and freely improvises roles.

The second part of the checklist is meant to evaluate a child’s self-regulatory behavior during joint play with peers. Gabeeva (2007), following theoretical ideas from B. D. El’konin, Zuckerman, A. L. Venger, and other cultural-historical scholars, formulated self-regulation criteria for primary school children. We modified Gabeeva’s criteria in line with the diagnostic methodologies developed by Galiguzova et al. (2013) for younger children. The second part of the inventory assesses the (3–6-years-old) child’s self-regulatory behavior during group play. Neuropsychologist Akhutina, head of the Neuropsychological Laboratory of Moscow State University, carried out an expert evaluation of the checklist.

Thus, this second part of the checklist also consists of seven parameters of a child’s self-regulatory behavior during joint play activity: (1) readiness to step into group play, (2) ability to coordinate one’s activities with those of other children, (3) amount of effort the child puts into creating a play, (4) ability to solve problems that arise during play, (5) child’s interest in joint play and their emotional regulation, (6) amount of adult assistance needed during play, (7) resistance toward external disturbances. Each parameter of self-regulatory behavior in play is ranked in four levels, from the lowest to the highest form of behavior, e.g., the amount of effort the child puts into creating a play:

– Does not develop play activity, often disrupts or stops the play.
– Offers no suggestions, accepts them from others, and continues playing.
– Offers suggestions, give orders to others, and continues playing.
– Offers suggestions that require their effort; takes others’ suggestions into account; develops the play further.

The *child’s play and self-regulation* checklist records the individual child’s skills only when they participate in joint play activity with peers. Not all play and self-regulation parameters can be observed in solitary play. As far as developed pretend role-play is concerned, it is a social role-play that is considered as such, as it creates optimal conditions for developing children’s play and self-regulation skills. Group play is usually of a collaborative nature and, therefore, a necessary condition for developing high-level play. The group of playing children acts about each participant of play as the organizing factor, sanctioning and supporting the performance of the role taken by the child.

### 3.5 Statistical data analyses

The IBM SPSS Statistics 26.0 software was used for statistical (quantitative) data analysis: descriptive analysis (frequencies, percentage rank, mean). The exploratory factor analysis (EFA) using the Principal Components Analysis with Varimax rotation was used to summarize the structure of a set of variables of the *child’s play and self-regulation checklist*. A Kaiser-Meyer-Olkin (KMO) test and Bartlett’s Test of Sphericity were first conducted to verify if the data set was suitable for factor analysis. The KMO index (0.958) and Bartlett’s test of sphericity (χ^2^ = 3359, 3799, *p* = 0.0001) indicated that the data were suitable for factor analysis. To verify the internal consistency of the checklist, we calculated Cronbach’s alpha. The *child’s play and self-regulation checklist* has a high rate of psychometric reliability: play part α = 0.907, self-regulation skills part α = 0.894.

To determine how the dependent variable (children’s self-regulation in play) is predicted by the independent variables (child’s gender, age, and play skills), a multiple regression analysis was performed. The data were also assessed for multicollinearity (variance inflation factor VIF < 4). The regression model was considered appropriate when the coefficient of determination *R*^2^ > 0.20 (Hair et al., 2019). A significance level of *p* < 0.05 was used for statistical analysis.

## 4 Results: Relationships between child’s play and self-regulation skills

In this section, we will present the study’s results, which aim to explore the relationship between the level of children’s play and self-regulation skills.

The study results of the 454 children show that *play* and *self-regulation* abilities are related. All relationships between these two variables are statistically significant, and most of them are of moderate strength. Correlation estimates are presented in Table 2.

**TABLE 2:** Correlations between the level of play and self-regulation skills (Kendall’s tau-b rank correlation coefficients).

SR criteria → Play level criteria ↓	Readiness to step into group play	Ability to coordinate one’s activities	Amount of effort the child puts into creating a play	Ability to solve problems that arise during play	Child’s interest in joint play and his emotional regulation	Amount of adult assistance needed during play	Resistance toward external disturbances	SR_rang_sum
Play objects	0.413***	0.374***	0.382***	0.398***	0.402***	0.458***	0.422***	0.476***
Self-position of the player	0.569***	0.511***	0.553***	0.495***	0.512***	0.528***	0.533***	0.641***
Interactions with a play partner	0.470***	0.489***	0.484***	0.468***	0.406***	0.438***	0.498***	0.551***
Play space	0.442***	0.374***	0.433***	0.374***	0.363***	0.465***	0.480***	0.502***
Play actions	0.487***	0.389***	0.502***	0.435***	0.429***	0.495***	0.475***	0.545***
Play plot	0.342***	0.368***	0.321***	0.328***	0.300***	0.409***	0.344***	0.419***
The main content of play	0.460***	0.393***	0.471***	0.433***	0.413***	0.447***	0.459***	0.539***
Play rang_sum	0.553***	0.475***	0.520***	0.482***	0.471***	0.546***	0.539***	0.638***

***p < 0.0001.

All play criteria are reliably related to the general self-regulation score, which shows children’s general self-regulation during play (Kendall’s correlation coefficients τ from 0.419 to 0.638). Analysis of the different play parameters showed that the *child’s position* in the play, i.e., the performance of the role during play activity, is mainly related to all self-regulation abilities and their overall assessment. The estimate of this play parameter with the overall self-regulation estimate is 0.641. The other four parameters–*interactions with a play partner*, *play space*, *play actions*, and *the main content of the play*, are more significant than 0.511, except for the correlations with the ability to manipulate the *play objects* (Kendall’s τ = 0.476) and *the play plot* (Kendall’s τ = 0.419). Thus, the research results confirm that the child’s behavior during play is controlled by their commitment to the assumed role, and this skill is related to the child’s self-regulation. It can be assumed that the higher the child’s play skills related to the level of the child’s position in the play, the higher the estimates of the child’s self-regulation skills will be. A child’s in-role play position requires him to follow specific rules related to self-regulation (e.g., the ability to coordinate one’s activities with those of other children or regulate one’s emotions, etc.).

The following three parameters of play are associated with medium-strength relationships with the child’s self-regulation: *the main content of play* (Kendall’s τ = 0.539), *play actions* (Kendall’s τ = 0.545), and *interactions with a play partner* (Kendall’s τ = 0.551). Attention should be paid to the last two–*the interactions of a play partner* and the *play actions*. The first sign that a child understands the rules of play and a role is when they start controlling play partners. In their play actions, the child sees specific models of role behavior and, telling a play partner how to behave, he “discovers” the rules of the role. Thus, compliance with the rules is closely related to the parameters of *interactions with a play partner* and *play actions*, which, according to the research data, is significantly related to the self-regulation skills of the playing child. The connections of other parameters, such as play objects, play space, and play plot, with the child’s self-regulation skills are weaker, although statistically significant.

Searching for connections between children’s pretend play and self-regulation, we also performed exploratory factor analysis with the principal component extraction method and Varimax rotation (Table 2). According to the two-factor model, the first factor consists of seven statements describing the child’s self-regulation during play. Their factor loadings range from 0.628 to 0.790. The second factor consists of statements describing the parameters of the child’s play. Their factor loadings vary from 0.595 to 0.814. This two-factor model explains 62.76 percent of variations (Table 3).

**TABLE 3:** Factor structure of the checklist for the evaluation of the level of play and self-regulation skill.

Items groups	Factors	
	1 (self- regulation)	2 (play)
Amount of effort the child puts into creating a play	0.790	
Child’s interest in joint play and his emotional regulation	0.770	
Ability to coordinate one’s activities with those of other children	0.754	
Ability to solve problems that arise during play	0.725	
Readiness to step into group play	0.723	
Resistance toward external disturbances	0.671	
Self-position of the child	0.666	0.459
Amount of adult assistance needed during play	0.628	
The play plot		0.814
Play objects		0.762
The main content of play		0.753
Play space		0.713
Play actions	0.465	0.606
Interactions with a play partner	0.513	0.595

It is necessary to note that the *self-position of the child* (play parameter) falls under both factors according to the factor analysis. In the first factor, the child’s self-regulatory behavior, its factorial weight is 0.666; in the second, in play, it is 0.459. As noted earlier, the role and the associated rules guide the child’s play actions, supporting their self-regulation. When “taking on” a specific role, the child embodies a particular behavior. In this way, the in-role position becomes a “key” that helps to understand the mechanism of formation of self-regulation in play. Therefore, the factor analysis only confirms this close connection when the child’s self-position (play parameter) also falls among the self-regulation criteria.

This first self-regulation factor includes the child’s *play actions* and *interactions with a play partner*, which reveals the importance of the participation of other children. However, their weights in the self-regulation factor are lower (0.465 and 0.513) than the play skills factor. However, it should be noted that these variables are also related to the child’s self-regulation process during role performance, and the factor analysis also illustrates this.

## 5 Results: The child’s play skills predict their self-regulatory behavior in play activity

Multiple linear regression analysis was performed to determine whether and how children’s play skills, age, and gender can predict children’s self-regulatory skills in play. Firstly, it shows no multicollinearity problem, as the VIF of all variables has a variance inflation factor of less than 4 (VIF < 4). The results of the regression model (Table 4) show that 69.6% of the dependent variable can be predicted by independent variables (*F* = 90.629, *p* < 0.0001).

**TABLE 4:** Multiple linear regression with children’s self-regulation skills in a play as dependent variable.

Independent variables	Standardized coefficients B	*t*	*p*-value	VIF
Constant	4.370	6.327	0.000	
Gender	−0.069	−2.255	0.025	1.081
Age	0.090	2.616	0.009	1.388
Play objects	0.074	1.687	0.093	2.277
Self-position of the player	0.329	6.977	0.000	2.611
Interactions with a play partner	0.149	3.308	0.001	2.390
Play space	0.072	1.597	0.111	2.389
Play actions	0.153	3.362	0.001	2.436
Play plot	−0.004	−0.104	0.918	2.192
The main content of play	0.141	2.993	0.003	2.601
F	90.629			
*p*	0.0001			
*R* ^2^	0.696			

For gender, 0 signifies “girl” and 1 “boy”.

Further improvement of the regression model resulted in retaining only statistically significant independent variables (Table 5). These are the child’s gender, age, and the five pretend play parameters (self-position of the player, interactions with a play partner, play space, play actions, and the main content of play). The resulting coefficient of determination (*R*^2^ = 0.681) showed a very slight decrease: 68.1% of children’s self-regulation skills in play can be explained by the child’s age, gender, and the five pretend play parameters.

**TABLE 5:** Multiple linear regression with children’s self-regulation skills in a play as dependent variable.

Independent variables	Standardized coefficients B	*t*	*p*-value	VIF
Constant	4.732	6.854	0.000	
Gender	−0.081	−2.710	0.007	1.042
Age	0.092	2.699	0.007	1.346
Self-position of the player	0.337	7.157	0.000	2.555
Interactions with a play partner	0.155	3.466	0.001	2.323
Play space	0.090	2.075	0.039	2.163
Play actions	0.157	3.430	0.001	2.431
The main content of play	0.152	3.335	0.001	2.394
F	112.435			
*p*	0.0001			
*R* ^2^	0.681			

For gender, 0 signifies “girl” and 1 “boy.”

The linear regression equation is written as follows:

Child’s self-regulation in play = 4.732 − 0.817G + 0.365A + 1.854SP + 0.910IP + 0.488PS + 0.827PA + 0.664MC

G, child’s gender; A, child’s age; SP, self-position of the player; IP, interactions with a play partner; PS, play space; PA, play actions; MC, the main content of play.

Thus, 68.1% of children’s self-regulation skills manifested in play are predicted by their play skills (self-position of the player, interactions with a play partner, play space, play actions, and the main content of play) together with the child’s gender and age. Of these predictors, the strongest predictor of self-regulation in play is the self-position of the player.

## 6 Discussion

It is well known that the theoretical framework defines the main concepts and research methods and allows conclusions and generalizations to be drawn, which can be made within the framework of the chosen theory. Our study defines the theoretical position very clearly. We underline that our tools, results, insights, and generalizations should be understood and interpreted in the context of cultural-historical and conceptually close theories. This is especially relevant to the concept and classification of children’s play activity and the interpretation of self-regulation, which we presented in the paper’s theoretical part.

From a cultural-historical theory perspective, high-level developed pretend play is only possible with the child’s self-regulation. From this theoretical perspective, we should discuss play as an *activity system*. Moreover, as with any human activity, play activity proceeds in two directions: it is turned to the external actions to construct the object of activity—play, and simultaneously, on the internal/mental actions that help to build the external object, joint play. So, a child at play is simultaneously the subject and the object of their activity, or as Kravtsov and Kravtsova (2010) and Kravtsova (2014) emphasized, the dual subject of play.

Thus, from the perspective of cultural-historical theory, our research only confirms the theoretical postulate. Regarding early childhood education praxis, which is often not based on specific theoretical perspectives and an understanding of early childhood development, the tool we have developed is important as it incorporates the child’s externally visible learning in the form of play actions, and the formation of their internal mental structures–self-regulation.

Children’s self-initiated play is significant from a developmental point of view because it is the first independent system of children’s activities in which they learn to agree on ideas and coordinate their actions while working toward satisfying result. Collaborative play is a very challenging activity that requires the maximum effort of the children and helps them to form structures of thought that are not possible when the child is playing individually or under adult guidance. These activities fundamentally differ because they help children develop different skills and thinking strategies. Children’s independent activities requiring collaboration are the most conducive to developing self-regulation.

The study’s results explore the relationship between the level of children’s play and self-regulation skills, confirming that *play* and *self-regulation* skills are related and that the relationships between these two variables are statistically significant. Analysis of the different play parameters revealed that the *self-position of the child* in the play, i.e., being in-role position during play activity, is related to all self-regulation parameters and their overall assessment.

Multiple linear regression analysis revealed that children’s self-regulation skills manifested in play could be predicted by five play parameters: the self-position of the player, interactions with a play partner, play actions, the main content of play, play space, and the child’s gender and age. The older the children, the higher their play parameters; boys showed slightly weaker play parameters than girls. Furthermore, again, the strongest predictor of self-regulation in play is the *self-position of the player*.

This is in line with El’konin (1999a,b, 2005a,2005b) and other scholars’ claim that *role*, together with the *play plot* (sjuzhet), is one of the main structural components of pretend role-play activity. Taking a play role is challenging for a young child. Being in a role requires the child to have at least a minimum level of self-regulation. More is needed for children to interact successfully with each other in their roles. Children in a role must monitor each other’s compliance with the rules of the role and co-construct the play events together, coordinating their actions and ideas.

Our previous study (Brėdikytė et al., 2015) found low play skills; only 30% of 3- to 6-year-old children achieve high-level play in each age group. This is confirmed by other researchers’ studies (Smirnova and Gudareva, 2004; Smirnova and Ryabkova, 2013; Ryabkova et al., 2017) and emphasized by many practitioners. Smirnova and Ryabkova (2013) performed a study that examined 112 children in the age group 5–6 years old in Moscow and revealed that only 3% of children showed a high level of pretend play.

The trend today, the decrease in play levels described in Ryabkova et al. (2017, 95) study, results in “children’s inability to create the imagined situation—to accept and hold the role, to develop a plot, or to create play space. Children cannot play meaningfully and peacefully during free play time—they are romping, fighting, and pushing. Moreover, instead of playing together, the teachers are filling the children’s free time with relaxing exercises or resorting to disciplinary actions.” This is a typical situation in a classroom where children are not used to playing regularly. The ability and desire to engage in pretend role play is not an inborn ability of children. Developing high-level play demands certain conditions, including adult participation and support. In today’s situation, the teacher’s competencies to understand play, support, and create play together with children are extremely important.

### 6.1 Limitations of the study

One of the limitations of our research might be that we study children’s self-regulation processes only in play activities and do not include everyday situations to see if the self-regulation acquired in play is transferred to other situations. It was a deliberate choice because, as already Vygotsky (2016, p. 18) pointed out: “In play a child is always above his average age, above his daily behavior; in play it is as though he were a head taller than himself.” Researching the transfer of acquired self-regulation skills to other life contexts requires a different research design for data collection and analysis. The role of adults in supporting transfer is critical, and they should also be included in the unit of such analysis.

The uniqueness of our study is that the teachers collected the data. There were many reasons for this, and we discussed them in the methodology section. We want to stress that this was a conscious decision reinforced after the researchers visited the kindergarten groups. Although our concern was that we would use video cameras and film the children, the children’s lives were disturbed by the appearance of two strangers in the group looking around, wondering, and sometimes seeming confused. After the first few visits, we realized we could not collect data ourselves. We wish to record the children’s highest achievements when playing with friends and expressing themselves freely. Who, if not the teacher, knows the children in her group best and can observe their play undisturbed? You cannot artificially create such situations, even less so for scientists who know children very little. We agree that this is a limitation of our study, but we realized that the researchers would not be able to capture authentic children’s play with just a few visits to the classroom. It should also be emphasized that we tried to ensure that the teachers assessing the children’s self-regulation and play skills had the same conceptual understanding of the phenomena. Cohen’s d (Kappa) criterion was used to analyze the data to determine the inter-rater reliability of the results (both teachers assessed the children in the group). In the case of extremely low Cohen’s d (Kappa) values (e.g., 0.209, 0.379), the second teacher’s ratings were not included in the analysis because they were unreliable. This result can be explained by the fact that these teachers did not participate in the project training. A qualitative analysis of their ratings showed that the teachers needed to understand the content of the criteria of play and self-regulation, their dynamics, and their interrelationships.

The choice (teacher or researcher) may have led to significant differences between our results and our Moscow colleagues. However, we believe that a child cannot unintentionally demonstrate the highest level of play and the lower level quickly. Therefore, the teachers only observed six children while collecting the data; each child was observed for at least 1 week, allowing the teacher to observe a child for a more extended period without rushing. We know the data teachers collected can only be considered partially accurate.

However, given that our study aimed to investigate the relationship between play and self-regulation rather than to measure and compare children’s abilities accurately, the fact that the trained teachers gathered the data from their classrooms does not diminish the relevance of the results. Still, this should be considered when interpreting the data from our study. Also, given that only 454 children and 75 teachers participated in our research, we must be cautious in making broader generalizations.

### 6.2 Implications for practice and further research

The child’s play and self-regulation (CP&SR) checklist is intended for ECEC professionals to understand better the structure of play activity and its key parameters. It is already used in Lithuania and included in university student training materials and teacher narrative play guidelines. The checklist can be a helpful tool to observe and assess children’s play and self-regulation skills. It also enables monitoring the development of the essential new formations of preschool age: imagination, elements of voluntary behavior, and symbolic function. Educators must recognize and understand the formation process of the child’s self-regulation.

The (CP&SR) checklist can be used for further research exploring children’s play and self-regulation. Further research in this area is needed to clarify how the self-regulatory skills of the young child are formed on an interpsychic level (between people) and how they become internalized and transferred to the child’s daily activities as a regular part of the human personality.

## 7 Conclusion

The study’s results suggest that children’s levels of play and self-regulation are statistically significantly related: the better a child plays, the higher their level of self-regulation. The results of the regression model show that 68.1% of children’s self-regulation skills manifested in play are predicted by their play skills, especially the following parameters of play: self-position of the player, interactions with a play partner, play space, play actions, the main content of play and the child’s gender and age.

## Data availability statement

The original contributions presented in this study are included in this article/Supplementary material, further inquiries can be directed to the corresponding author.

## Ethics statement

The studies involving humans were approved by the Vytautas Magnus University Education Academy Ethics Committee has approved the study. The studies were conducted in accordance with the local legislation and institutional requirements. Written informed consent for participation in this study was provided by the participants’ legal guardians/next of kin.

## Author contributions

MB: conceptualization and writing—review and editing. MB and AB: methodology, investigation, and writing—original draft preparation. AB: formal analysis. All authors have read and agreed to the published version of the manuscript.
