# Supplementary material for: Pretend play as the space for development of self-regulation: cultural-historical perspective
Source: Front Psychol. 2023 Dec 19;14:1186512. doi: 10.3389/fpsyg.2023.1186512 (PMC10766374; doi:10.3389/fpsyg.2023.1186512)
Supplement: Supplementary file 1 [file Data_Sheet_1.docx]

Supplementary Material

**Pretend Play as the Space for Development of Self-Regulation: Cultural-Historical Perspective**

Bredikyte Milda*, Brandisauskiene Agne

*** Correspondence:** Bredikyte Milda: bredikyte.milda@vdu.lt

**Appendix 1**

**Child’s Play and Self-Regulation Checklist (CP&SR)** (3-6-years-old*)*

Child’s initials

Child’s age

Child’s gender

Child’s group/class

1. **Level of child’s play.** *Evaluate each statement (1-7) by selecting the most appropriate answer (a-d) and marking X in the box:*

| 1. | **Play objects** | *X* |
| --- | --- | --- |
| a | Child uses objects according to their intended purpose |  |
| b | Child plays with real objects, sometimes with substitutes |  |
| c | Child plays with substitute objects, sometimes with imaginary objects |  |
| d | Child plays with imaginary objects |  |
| **2.** | **Self-position of the child** |  |
| a | Child has no role |  |
| b | Child has a role but does not keep to the rules of the role or is inconsistent |  |
| c | Child has a role and keeps to the rules of the role |  |
| d | Child is flexible and freely improvises roles |  |
| 3. | **Interactions with a play partner** |  |
| a | Child’s play partner is real, has no role, no in-role interactions |  |
| b | Child’s play partner has a role, but very few interactions between the players |  |
| c | Child's play partner’s actions are determined by the role, many in-role play interactions |  |
| d | Many in-role play interactions with a play partner, but also partner may be imaginary |  |
| 4. | **Play space** |  |
| a | Play space is real |  |
| b | Play space is real, purposefully chosen |  |
| c | Play space is created/constructed |  |
| d | Play space is created and may be imagined, only marked by words and/or actions |  |
| 5. | **Play actions** |  |
| a | Child performs separate play operations (feeding a doll). |  |
| b | Child performs separate play actions (combines 2-3 operations: undresses doll, puts it to bed, covers it) |  |
| c | Child creates and sustains a schematic play event (birthday party, visiting a doctor, building a house, flying in an airplane etc.) |  |
| d | Child creates a successive chain of play events (flexibly combining play events in a continuing storyline) |  |
| 6. | **The play plot (sjuzhet)** |  |
| a | Repetitive realistic daily events |  |
| b | Improvised daily life episodes |  |
| c | Adventures |  |
| d | Fantastic / fictional events |  |
| 7. | **The main content/motive of play** |  |
| a | Actions with objects |  |
| b | Role actions (determined by the chosen role) |  |
| c | Interactions with partner(s) |  |
| d | Creation of play events and construction of play plot/narrative |  |

1. **Child’s self-regulation during group play.** *Evaluate each statement (8-14) by selecting the most appropriate answer (a-d) and marking X in the options box:*

| 8. | **Readiness to step into group play** | *X* |
| --- | --- | --- |
| a | Does not join into play |  |
| b | Observes play of others; joins after a few suggestions; can play next to others; or play only with regular play partner(s) |  |
| c | Joins in the play if accepted, usually plays with regular play partner(s) |  |
| d | Joins in right away, using effective strategies, plays with different groups of children |  |
| **9.** | **Ability to coordinate one’s activities with those of other children** |  |
| a | Does not take others’ activities into account |  |
| b | Takes others’ activities into account and tries to change the play situation to his benefit |  |
| c | Coordinates his activities to those of others, sometimes considering his interests, sometimes those of others |  |
| d | Coordinates and adjusts their actions according to the course of the play |  |
| 10. | **Amount of effort the child puts into creating a play** |  |
| a | Does not develop play activity, often disrupts or stops the play. |  |
| b | Offers no suggestions, accepts them from others, continues playing. |  |
| c | Offers suggestions, gives orders to others, continues playing. |  |
| d | Offers suggestions that require their own effort; takes others’ suggestions into account; develops the play further. |  |
| 11. | **Ability to solve problems that arise during play** |  |
| a | Sticks to their own position and disagrees with others' suggestions |  |
| b | Accepts others’ suggestions |  |
| c | Accepts others’ suggestions or offers their suggestions, encouraging himself and others to give up their interests in favor of joint play |  |
| d | Takes the initiative to solve a problem, finding a solution that satisfies everyone in the group. |  |
| 12. | **Child’s interest in joint play and their emotional regulation** |  |
| a | Not interested in joint play (does not observe, does not imitate), does not establish contact with other players. |  |
| b | Shows interest, sometimes plays (joins and leaves the play), may have a strong emotional reaction (shyness, joy) or in case of unexpected interference, an abrupt emotional outburst (shouting, throwing objects, hitting, biting, leaving the play). |  |
| c | The child is interested in the play, displays more joyful than negative emotions. In case of unexpected interference, negative emotions reactions may occur (irritability, raised voice, pulling away), but not be expressed in physical aggression. |  |
| d | The child is very involved in play activity (tuned into the role, the play events), displays joyful emotions, calmly and flexibly responds to unexpected interference, notices others’ emotions, can show joy together with others, or try to encourage and reassure them. |  |
| 13. | **Amount of adult assistance needed during play** |  |
| a | Plays only under the guidance of an adult. |  |
| b | Can play independently, performs play actions, and often asks for help when faced with an obstacle. |  |
| c | Tries to find own solution, asks for help only if a solution cannot be found. |  |
| d | Organizes and develops play activity on their own. |  |
| 14. | **Resistance towards external disturbances** |  |
| a | Due to an external disturbance, the child's play is interrupted or transformed into another activity. |  |
| b | The play becomes fragmented and inconsistent when an external disturbance occurs, and external (adult) support is needed to keep the play going. |  |
| c | Due to the external disturbance, the child interrupts the play for a short time but later returns to it and continues to play. |  |
| d | External disturbances do not stop the play and do not affect its quality. A child can use specific strategies for the continuation of the play. |  |
